# Supplementary material for: Testicular toxicity following chronic codeine administration is via oxidative DNA damage and up-regulation of NO/TNF-α and caspase 3 activities
Source: PLoS One. 2020 Mar 13;15(3):e0224052. doi: 10.1371/journal.pone.0224052 (PMC7069647; doi:10.1371/journal.pone.0224052)
Supplement: Attachment — Submitted filename: PONE, Response to reviewers REAL.docx [file pone.0224052.s004.docx]

**Sir/ma,**

**REBUTTAL LETTER TO COMMENTS BY EDITOR AND REVIEWERS**

The following comments raised by the academic editor on the original manuscript should be addressed.

Additional Editor Comments (if provided):

The testicular toxicity due to drug abuse is well reported in many studies. However, the effect of codeine on testicular toxicity is not well understood and this manuscript provides basic on this aspect.

Additional experiments that provides an in depth analyses on the molecular and cellular mechanisms of codeine toxicity will add more strength to the study.

**Response: Thanks. More molecular and cellular mechanisms to buttress codeine-induced testicular toxicity has been added such as testicular DNA fragmentation and testicular TNF-α**

Statistical analyses are confusing and need to be carefully carried out. In many instances, there appears to be no significance, but it is indicated as significant.

**Response: Thanks. This has been clarified and well stated in the manuscript**

Fructose is the main source of energy for spermatozoa. The rationale in measuring glucose is not provided. How does this fit?

**Response: Thanks. Although fructose is hypothesized as the main energy source of sperm, lactate remains the preferred energy substrate which is primarily derived from glucose, and partly from fructose, via the glycolytic pathway. A detailed explanation with references has been included in the manuscript.**

Concrete evidence should be provided as to why testosterone decreases due to codeine treatment? Is it because of the altered levels of FSH / LH or any other cause?

**Response: Thanks. Concrete evidences have been clearly explained in the DISCUSSION section, and a summary in the CONCLUSION.**

**Codeine depletes testicular glutathione, SOD, and catalase resulting in increased ROS production and inflammatory response which in turn leads to testicular lipid peroxidation, protein denaturation/inactivation, and testicular DNA damage, with consequent caspase-mediated testicular apoptosis and loss of testicular function**.

Anti-oxidant enzyme activities should be presented as U/mg protein and not U/ml. Else it will be difficult to normalize the activities / levels between the samples.

**Response: Thanks. This has been incorporated.**

Caspase-3 activity should be presented, not the levels (ng/ml).

**Response: Thanks. This has been incorporated.**

**Thanks for the comments. They added value to the manuscript.**
